# Supplementary material for: RNA-Seq Dissects Incomplete Activation of Phytoalexin Biosynthesis by the Soybean Transcription Factors GmMYB29A2 and GmNAC42-1
Source: Plants (Basel). 2023 Jan 25;12(3):545. doi: 10.3390/plants12030545 (PMC9921300; doi:10.3390/plants12030545)
Supplement: Supplementary file 1 [file plants-12-00545-s001.zip › Supplemental Figure S1. Ontologies of co-regulated genes..pdf]

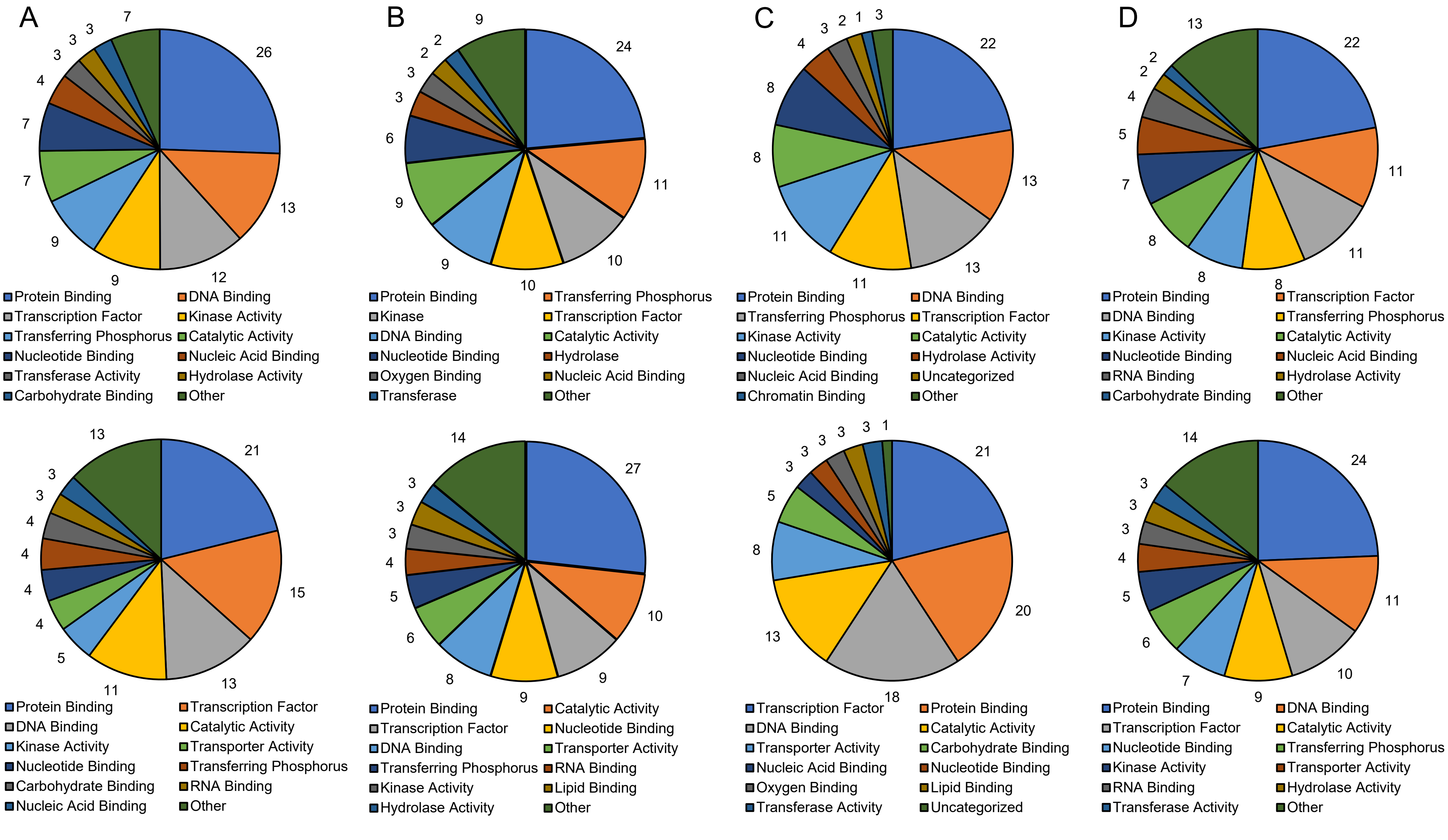

Supplemental Figure S1. Ontologies of genes co-regulated and exclusively regulated by WGE. Upregulated (top) and downregulated (bottom) percent ontologies are shown. (**A**) By WGE and *p35S::GmNAC42-1*. (**B**) By WGE and *p35S::GmMYB29A2*. (**C**) By WGE, *p35S::GmNAC42-1, and p35S::GmMYB29A2*. (**D**) By WGE only. Ontology analysis was conducted using the SoyBase.org (Accessed on 20 December 2022) Gene model Data Mining and Analysis tool.
