# Supplementary material for: RNA-Seq Dissects Incomplete Activation of Phytoalexin Biosynthesis by the Soybean Transcription Factors GmMYB29A2 and GmNAC42-1
Source: Plants (Basel). 2023 Jan 25;12(3):545. doi: 10.3390/plants12030545 (PMC9921300; doi:10.3390/plants12030545)
Supplement: Supplementary file 1 [file plants-12-00545-s001.zip › Supplemental Figure S2. Similarity and identity among GmMYB29A2 and VvMYB14 amino acid sequences..pdf]

A

```
                                     Repeat R2

GmMYB29A2    MVRAPCCEKMGLKKGPWTPEEDQILMSYIQKHGHGNWRALPKLAGLLRCGKSCRLRWINY  60
VvMYB14      MGRAPCCEKMGLKKGPWTPEEDQILVNYIHLYGHGNWRALPKQAGLLRCGKSCRLRWTNY  60
             * ***********************:.**: :********** ************** **

                             Repeat R3

GmMYB29A2    LRPDIKRGNFSSEEEEIIIKMHELLGNRWSAIAAKLPGRTDNEIKNVWHTHLKKRLLNSD  120
VvMYB14      LRPDIKRGNFTSEEEETIIELHERLGNRWSAIAAKLPGRTDNEIKNVWHTHLKKRLKHNH  120
             **********:***** **::** ******************************** :..

GmMYB29A2    IQKRVSKPRIKRSDSNSSTLTQLEPTSSA---------CT------TSLSDFSSFSE---  162
VvMYB14      ATPPPKRHSLDASQ----VEKQQNPINSATNSRSESLGYGPVLSPQPSFSDISSAATTTT  176
                  .:  :. *:    . .* :* .**                  *:**:** :

GmMYB29A2    --GTKNMDNMIKSEDIESVETIMPPIDESFWSEATVDYESSTMMTSNSWTISNELAPPQY  220
VvMYB14      TTTTATMSDITTPCIKVDSPEDFPEMDENFWSEVLSSNKSGAAG-----DLPGAASGPQL  231
                * .*.:: .     .    :* :**.****.  . :*.:        : .  : **

GmMYB29A2    QFNSVESFQQQSVDYNGSNDDHDGMDFWYDIFIKSGESIELPEF 264
VvMYB14      QFPFSPRAVIGSSPY---STYDMDMEFWYNIFTRSGELHELSEI 272
             **         *  *   .  . .*:***:** :***  ** *:
```

```
Percent identity: 49.65
Percent similarity: 54.58
```

B

```
                           Repeat R2

GmMYB29A2    LKKGPWTPEEDQILMSYIQKHGHGNWRALPKLAGLLRCGKSCRLRWINYLRPDIKRGNFS  60
VvMYB14      LKKGPWTPEEDQILVNYIHLYGHGNWRALPKQAGLLRCGKSCRLRWTNYLRPDIKRGNFT  60
             **************:.**: :********** ************** ************:

                        Repeat R3

GmMYB29A2    SEEEEIIIKMHELLGNRWSAIAAKLPGRTDNEIKNVWHTHLKKRL        105
VvMYB14      SEEEETIIELHERLGNRWSAIAAKLPGRTDNEIKNVWHTHLKKRL        105
             ***** **::** ********************************
```

```
Percent identity: 88.57
Percent similarity: 89.52
```

Supplemental Figure S2. Similarity and identity among GmMYB29A2 and VvMYB14 amino acid sequences. (A) Full-length amino acid sequences. (B) R2 R3 putative DNA-binding domain sequences. '*' indicates identical amino acids, ':' amino acids with similar biochemical features.
